# Digital trajectories of acculturation: social media practices among a sample of African female domestic workers

**DOI:** 10.3389/fsoc.2026.1854813

**Published:** 2026-08-17

**Authors:** Najia Khamis, Samar Ben Romdhane, Sang Lee

**Affiliations:** College of Mass Communication, Ajman University, Ajman, United Arab Emirates

**Keywords:** acculturation, African diaspora, African migrant domestic workers, gendered labor, qualitative research

## Abstract

Globalization and cross-cultural migration have intensified the movement of individuals across national and cultural boundaries, particularly among migrants employed in feminized labor sectors. Within these contexts, acculturation is shaped not only by face-to-face interactions but also by engagement with digital media. This study examines how and why African female domestic workers in the United Arab Emirates (UAE) use social media and how these practices shape their acculturation experiences. Using a qualitative research design, the study draws on in-depth interviews with African female domestic workers from Ethiopia, Uganda, and Nigeria. The data were analyzed using thematic and narrative analysis to identify recurring patterns while preserving participants’ individual lived experiences. Findings indicate that social media serves multiple functions in participants’ acculturation processes. It enables them to sustain transnational family connections, learn about host-country cultural norms and workplace expectations, access emotional and spiritual support, and maintain religious identities and collective cultural values. At the same time, participants’ selective and cautious engagement with digital platforms reflects tensions within these practices, as social media can facilitate connection and cultural adaptation while also contributing to digital stress. Overall, the findings position social media as a multidimensional mediating space through which migrant domestic workers negotiate cultural learning, belonging, identity, and transnational relationships. The study contributes to migration and digital media scholarship by demonstrating that social media can simultaneously facilitate and complicate acculturation among an understudied population of African female domestic workers in the UAE.

## 1 Introduction

Within Asia, significant movements of migrant labor from countries such as Indonesia, India, Bangladesh, and the Philippines to Singapore, Hong Kong, and Gulf countries have been documented (eg, Sweileh, 2024). A similar labor migration trend has emerged from African countries, with workers from Kenya, Ethiopia, and other parts of Africa coming to the UAE to fill demand in domestic work. These movements reflect broader global patterns of labor mobility that have intensified among women, a phenomenon described as the feminization of migration (Sassen-Koob, 1984; Sweileh, 2024).

Female migrant domestic workers, primarily performing household tasks (Sweileh, 2024), represents one of the largest categories of female migrant labor (Donato and Gabaccia, 2015). While it mirrors global inequalities and gendered labor hierarchies (Kutlu and Karataş, 2025), migration for these women, can paradoxically provide opportunities for increased autonomy and empowerment, helping them to break the chain of precarity (Silvey and Parreñas, 2020) and shifting power dynamics within their families and communities. As demonstrated in many studies, women working as domestic workers exercise agency to deal with the power dynamics inherent to paid domestic labor (Ali et al., 2025). This agency can take multiple forms, such as leveraging social and digital networks for support, adaptation, and acculturation. As this form of work is situated within the private sphere, acculturation possibilities are shaped by the limited exposure to the broader external environment. In this context, social media may function as a mediated space enabling belonging to an occupational community (Golan and Babis, 2019) cultural exposure, and transnational connectivity (eg., Roosen et al., 2021).

This broader perspective on domestic work as both gendered labor and a potential avenue for broader economic benefits as well as individual empowerment becomes particularly tangible when examining the specific context of the UAE. The UAE hosts a substantial number of migrant domestic workers, primarily from Africa and South Asia (Ewers and Dicce, 2016). Recognizing the vulnerabilities inherent to this type of work, the UAE government implemented measures supporting overseas domestic workers with a legal framework that governs domestic work (Federal Law Number 10 of 2017). The law guarantees rights to written contracts, age restrictions, guarantee for paid weekly rest days, annual leave, health coverage, and accommodation. Reflecting an effort to institutionalize the sector, the Ministry of Resources and Emiratization (MOHRE), oversees their recruitment and employment.

The wave of migration into the UAE over the last couple of decades has coincided with the emergence of digital and social media platforms, which have become essential tools for migrant workers’ acculturation. The UAE is recognized for its high Internet penetration and fast connectivity, with social media usage among the highest in the world (International Telecommunication Union, 2023), as well as the widespread social media engagement (Baycar, 2024). For foreign workers, these platforms provide opportunities to connect with their home countries and sustain social interactions, access information on local norms and expectations, and preserve their original heritage, balancing integration and cultural continuity during the acculturation process (Berry, 1997). Domestic workers in UAE need to “observe the customs and traditions of society and adhere to public morals” (Federal Decree-Law No. 9 of 2022 Concerning Domestic Workers, United Arab Emirates, 2022, art.12), which may balance he use of digital tools for personal empowerment with respect for the sociocultural frameworks of the UAE.

According to Berry (1997) proposal, there are four main acculturation strategies: integration, assimilation, separation, and marginalization. Integration refers to maintaining one’s heritage culture while engaging with the host society, whereas assimilation involves abandoning cultural maintenance in favor of host-society adoption. Separation reflects strong maintenance of the home culture with limited engagement in the host environment, while marginalization describes a weakened orientation toward both cultural contexts. These strategies provide an analytical lens for understanding how migrant workers negotiate cultural continuity and adaptation in digitally mediated environment. From a Uses and Gratifications Theory (UGT) perspective, migrants are not passive media consumers but active users who select media to satisfy specific psychological and social needs (Katz et al., 1973). In the context of migrant domestic work, social media use may be understood through multiple gratifications, including informational needs (e.g., workplace learning and cultural adaptation), relational and emotional needs (e.g., family connection and social support), and spiritual needs (e.g., religious engagement and identity reinforcement). These gratifications are not only outcomes of media use but may also function as mechanisms that shape acculturation trajectories.

Although migrant workers have been present in many countries for decades, the role of social media in facilitating the acculturation of female domestic workers has received limited scholarly attention.

Although research has examined social media use in migrant adaptation and acculturation processes, it has primarily focused on international students, highly skilled migrants, and diaspora communities, particularly in Western contexts. More recently, scholars have begun to explore the intersection of social media and acculturation among migrant populations (Hofhuis et al., 2023; Chen and Li, 2023; Lim and Pham, 2016). Despite these developments, limited attention has been paid to low-income, live-in domestic workers in the Gulf region, whose migration experiences are shaped by unique structural conditions, including restricted mobility, and employer-dependent living arrangements. Consequently, little is known about how African female domestic workers in the United Arab Emirates use social media as a resource for managing transnational family obligations, negotiating emotional and spiritual well-being, and adapting to the host environment.

In parallel, research on migrant domestic workers has predominantly addressed issues of labor precarity, gendered exploitation, and mobility constraints, with limited attention to their everyday digital practices. In particular, there remains a lack of understanding of how specific media gratifications (e.g., informational, emotional, relational, and technological) interact with acculturation processes in this context. This study addresses this gap by examining how social media use among African female domestic workers in the UAE is embedded within broader cultural orientations, migration constraints, and everyday survival strategies, and how these dynamics shape distinct acculturation trajectories.

## 2 Literature review

Literature in migrant studies indicates that communication functions as a means of coping with cultural differences, isolation, and stress (e.g., Akter et al., 2024; Berry, 1997). Migrants rely on digital platforms to build networks, negotiate identity, and advocate for their rights (Mitra et al., 2026). Consequently, the use of digital platforms, affects migrants’ well-being and the maintenance of their identity across borders (Lintelo et al., 2024).

Migrants commonly face challenges such as language barriers, cultural dissonance, and limited social networks, which may lead to negative consequences, such as psychological distress and social isolation (Kim et al., 2022). In this context, social media can serve as a symbolic space for identity negotiation and reconstruction (Yau et al., 2020), enabling migrant workers to maintain ties with their heritage cultures while simultaneously engaging with the host society (Akter et al., 2024; Baycar, 2024; Chen and Li, 2023).

### 2.1 Beyond cultural essentialism: collectivism and digital media practices among migrant workers

Cultural orientation among migrant workers may shape how they engage with digital platforms and the types of gratifications they seek from them. Prior research suggests that African migrant domestic workers often come from contexts broadly characterized as collectivist, where social harmony, familial obligations, and social interdependence are more emphasized than individual expression and uniqueness (Zhao, 2025). However, African societies are diverse, and collectivist orientations should not be interpreted as uniform across the continent. For instance, South Africa exhibits a “dualistic” value system that challenged the traditional collectivist narrative (Coldwell et al., 2023). This study therefore recognizes the importance of avoiding monolithic representations of “African culture” and adapts a more nuanced approach that focuses on lived experiences of participants analyses, taking into account variations in their social conditions, trajectories, beliefs, and variations in their original countries.

Hofstede describes collectivism as cultural pattern in which individuals are integrated into strong, cohesive groups that offer protection in exchange for loyalty (Hofstede, 1980; Hofstede, 2011). However, Hofstede’s cultural dimensions and the assumption of stable, nation-level value consensus have been widely criticized for cultural essentialism and for overlooking internal diversity and variations (Vaiman and Taras, 2026). In response to these limitations, collectivism, in this study is not treated as a fixed national attribute, but rather as a set of dynamic and relational practices, enacted within diasporic and digitally mediated contexts, and shaped by migration experiences, religious beliefs, structural conditions of labor precarity, and other contextual factors (Vaiman and Taras, 2026).

Empirical studies indicate that societies like Ethiopia and Uganda are often characterized by relational orientations in which extended family structures are central and a “we” perspective is privileged over an individualistic “I” perspective (eg, Bukuluki, 2013; Nigatu, 2018). In migration context, however, these orientations are not simply transferred stable and intact. Rather they are continuously reconfigured through transnational practices and digital communication experiences.

In collectivist-oriented relational settings, digital platforms are not only used for individual expression, but are strategically mobilized to fulfill relational and emotional gratifications, particularly the maintenance of family ties and social belonging across distance. These cultural orientations therefore influence communication practices, including how individuals exchange information, maintain social relationships, and negotiate social belonging (e.g., Chee, 2023; Chen and Li, 2023). In particular, collectivist orientations are reflected in the strong emphasis on social relationships and social group cohesion, which is reflected in patterns of social media use. Individuals from collectivist context use social media to mainly reinforce their relationships with their families, friends, and their social networks in general (Hofstede, 2011), thereby satisfying key UGT dimensions such as emotional support, social connectivity, and identity reinforcement.

In migration settings, social media, such as messaging apps and social networking sites, allow individuals to maintain close and frequent interaction with their close associates, relatives, and members of their social networks. Research shows that migrants and individuals from collectivistic cultures use social media to sustain transnational family relationships and strengthen emotional bonds with their loved ones, despite their physical inaccessibility (Chen and Li, 2023).

Another prominent feature in this regard is the role of social media in facilitating social support and cooperation. In collectivist societies, individuals are expected to help members of their social groups, especially during adverse situations. Social media sites can be used as a means for seeking advice or support from members of social groups. For instance, migrant workers often seek information through social media groups about employment conditions or housing in the host country (e.g., Akter et al., 2024). Such groups can be regarded as a support system for members of a certain culture or community. Collectivist values can be seen in the way individuals from these cultures express themselves on social media platforms. Individuals from collectivist cultures tend to be more careful in expressing their opinions on social media sites in a way that would lead to conflict within their social groups. Rather than expressing their individual opinions or achievements on social media sites, individuals from these cultures often share information on social media sites that focus on activities conducted within groups or families (Kim et al., 2022).

Furthermore, collectivistic cultural values influence the formation of online communities based on shared identities like nationalities, ethnicities, religious affiliations, and professional associations. Through these online communities, people can maintain their cultural practices, disseminate content based on their languages, and uphold collectivistic values while living in different cultural environments. In the case of migrants, these online communities help alleviate the sense of loneliness and maintain cultural continuity. Thus, the role of social media is not only restricted to facilitating communication but is also related to maintaining cultural continuity and group identities across physical spaces (Brinkerhoff, 2009).

Within an acculturation framework, these digitally mediated interactions also facilitate adaptation processes by enabling migrants to remain connected to their home culture while gradually engaging with host-society norms. Accordingly, social media functions simultaneously as a gratification-seeking environment and an acculturative resource, supporting both continuity with the home culture and adjustment to the host context.

### 2.2 Uses and gratifications of social media for migrant workers

UGT provides a motivational framework to examine why and how users actively select and use specific media to satisfy their needs (Katz et al., 1973). Gratifications can be categorized according to the psychological, social, and functional needs they fulfill and may include informational, social, emotional, affordance-related gratifications (Wang, 2021).

For migrants, social media use is often driven by the need for emotional reassurance, social support, and practical guidance relevant to their everyday challenges (Akter et al., 2024; Calvo et al., 2024; Komito, 2011). In the case of African female domestic workers, choosing a communication platform is not merely a technical preference but a reflection of their psychological and emotional needs. For example, those who experience homesickness may join Facebook or Telegram groups to remain emotionally connected to family and friends, helping them maintain family ties and a sense of cultural identity. African female domestic workers may also seek knowledge and information online to better understand the host country environment. Further, social media platforms can be essential tools to express self for female domestic workers, whose social interactions are often restricted by long working hours, live-in arrangements, and limited access to public spaces. To understand domestic workers’ motivations and gratifications related to media use, UGT perspectives offer a nuanced lens to elicit how social media uses intersects with acculturation, identity negotiation, and well-being in transnational settings (Akter et al., 2024; Chen and Li, 2023; Ruggiero, 2000).

Research has demonstrated that social media plays a crucial role in enabling migrant workers to maintain ties with their home cultures while simultaneously facilitating acculturation into the host society, a process described as bidirectional or hybrid identity formation (Calvo et al., 2024). While social media can be helpful in learning the language, cultural practices, and religious content of the host country, various social media platforms also help migrant workers update family events and home country news, allowing them to reaffirm or sustain their cultural heritage and resist identity erosion caused by displacement and marginalization (Akter et al., 2024; Calvo et al., 2024). At the same time, exposure to both host and home countries’ cultural norms, values, and practices through digital content allows migrant workers to gradually negotiate new social roles and identities (Akter et al., 2024).

For female domestic workers, social media use and interactions are particularly significant because their offline social worlds are often limited by workplace requirements and structural conditions. Social media platforms can offer opportunities for self-representation, emotional expression, and collective identity-building that may not be possible in their physical environments due to the demands of their job duties in the host country (Hurley, 2023; Komito, 2011). Therefore, these platforms function as arenas of belonging, where migrant workers construct a sense of “being seen” and recognized, even when socially isolated and invisible in host societies (Yau et al., 2020). At the same time, it should also be noted that while digital spaces can enable migrant workers, they may also further isolate workers from the outside world and reinforce inward-looking identities that limit broader social integration into the host country (e.g., Mitra and Evansluong, 2019).

### 2.3 From gratification to acculturation among migrant workers

While previous studies have examined social media use and acculturation as related phenomena, this study draws on UGT to conceptualize gratifications as mechanisms that shape the direction and mode of acculturation.

Berry’s acculturation model identifies four primary strategies that individuals or groups adopt when adapting to a new culture. These strategies are based on two dimensions: the degree to which individuals maintain their heritage culture and the extent of their engagement with the host culture.

Specifically, Informational gratifications, such as learning workplace norms or local customs, facilitate integration by equipping workers with cultural knowledge necessary for participation in the host society. Relational and emotional gratifications, such as transnational family connection and diaspora solidarity, may reinforce separation or ethnic maintenance by anchoring identity in the home culture. This perspective provides a theoretical bridge between migrants’ motivations for social media use and the acculturation outcomes explored in this study.

Some researchers have noted that the relationship between social media and acculturation is complex because it can amplify power dynamics and struggles within immigrant communities, particularly among domestic workers (Mitra and Evansluong, 2019; Chen and Li, 2023). Others elaborate on how these digital interactions drive acculturation and enhance social opportunities (Akter et al., 2024; Pang, 2020). However, research generally suggests that social media facilitates acculturation by providing platforms for connection and engagement (Akter et al., 2024; Baycar, 2024). Social media platforms enable users to form acquaintances, build relationships, incorporate mainstream cultural behaviors, and participate in intergroup discourse that supports acculturation.

Due to their hybrid nature, social media platforms foster an environment that helps migrants negotiate and reshape cultural practices by encouraging them to reconcile between host- and home-country cultures (Chen and Li, 2023). Such use of social media promotes identity integration and self-concept by increasing confidence in using the host language and culture (Chen and Li, 2023). Social media sustains cultural values and practices, facilitates sharing and identity development, and shapes social media habits (Brinkerhoff, 2009). Through constant interactions, these platforms support identity integration and reinforce self-concept, while also helping to protect cultural values and customs, contributing to cultural identity exchange (Komito, 2011).

For migrant workers, social media platforms can function as a means of building community and negotiating personal and collective identity in an often unstable social and legal context (Hurley, 2023). These platforms also facilitate emotional sustenance, resistance, and patience in enduring working conditions and separation from home, and enable dialogue, storytelling, and collective resilience. Importantly, while most studies suggest a positive role of social media for the acculturation of migrant workers, studies show that digital platform design plays a dual role; it can allow users to express themselves while also imposing indirect constraints (Alencar, 2018). For example, the algorithms of social media platforms may result in exposure to selective media content, limiting exposure to broader cultural and social contexts in the host country (Hartmann et al., 2025). Further, for those domestic workers whose work conditions are confined to their living spaces, social media may be used as a tool to escape from harsh realities and thereby further isolate themselves (Chen and Li, 2023; Brinkerhoff, 2009).

Based on the review thus far, the following research questions were developed to explore how social media platforms influence the acculturation of a sample of female African domestic workers in the UAE:

RQ1: How do cultural orientations shape African domestic workers ‘engagement with social media in the acculturation process?

RQ2: How do African domestic workers social media practices shape the relationship between their social, emotional, and informational needs and their acculturation experiences?

## 3 Method

This study explores how a sample of African domestic workers in the UAE use social media to navigate their cultural adaptation and identity formation. It focuses on their narratives, social interactions, and digital experiences.

### 3.1 Research design

Given the exploratory nature of the research questions and the limited scholarly attention to this population (i.e., African domestic workers in the UAE), this study adopts a qualitative design using In-depth interviews. The study is situated within an interpretive epistemological stance, which conceptualizes social reality as socially constructed through individuals’ lived experiences and situated within broader socio-cultural and migratory contexts.

The study employs qualitative approach to guide the analytical orientation of the research. This approach is suited to exploring how participants make sense of their social media practices in relation to acculturation, identity, and everyday life in temporary labor migration.

### 3.2 Recruitment and participants

The selection of a sample of African domestic workers reflects recent shifts in labor migration patterns in the UAE, with increasing recruitment from Ethiopia and Kenya. Despite this growing presence, African domestic workers remain understudied compared to Asian workers. Focusing on this group, participants were recruited through a snowball sampling method via neighbors and community contacts. This sampling method was considered ideal because the demographic information of domestic workers was not accessible due to privacy regulations in the UAE. The employers were contacted first to obtain permission for interviews with their domestic workers. Two essential sampling guidelines were used to recruit participants. First, participants had to be female domestic workers from African countries. Second, participants were required to have experience with social media, as disclosure of usage of social media. Usage was necessary to explore its role in acculturation, even though it is acknowledged that some individuals may not disclose its usage. Twelve female domestic workers were recruited for interviews (see Table 1 for participants’ demographic information). The interviews were conducted in the private rooms of their workplaces to ensure confidentiality and comfort. Participants, aged between 20 and 30 years old, were drawn from three nationalities, including Ethiopian, Ugandan, and Nigerian, with a majority being Ethiopian. While this composition reflects current recruitment patterns in domestic labor, it also limits the diversity of perspectives captured. Accordingly, the study does not claim representativeness of African domestic workers as a whole, but rather focuses on in-depth exploration of participants’ lived experiences, social media practices, and acculturation strategies within this specific context. Most of them had an education level between Grades 3 and 10. They had household working experience ranging from nine months to eight years in the UAE. Six of them worked primarily as cooks, and the others were responsible for cleaning and other household chores. Six of the participants were married, five were single, and one was divorced.

**Table 1:** Participants’ demographic information.

Participants	Age	Nationality	Education level	Years in the UAE	Role/Type of work	Interview language
P1	20	Ethiopia	3	5	Cooking	Arabic
P2	25	Ethiopia	3	8	Cleaning/laundry	Arabic
P3	28	Ethiopia	7	2	Cleaning/laundry	Arabic
P4	23	Ethiopia	10	9 months	Cooking/laundry	Arabic/English
P5	23	Ethiopia	5	3	Cleaning/laundry	Arabic
P6	25	Ethiopia	5	8	Cooking /laundry	Arabic
P7	28	Ethiopia	10	3	Cooking/cleaning	Arabic/English
P8	26	Ethiopia	7	4	Cooking/cleaning	Arabic/English
P9	30	Uganda	10	4	Cleaning/laundry	English
P10	23	Nigeria	3	4	Cleaning/baby care	English
P11	27	Ethiopia	7	5	Cooking/laundry	Arabic
P12	23	Ethiopia	10	5	Cleaning/laundry	Arabic

### 3.3 Data collection and analysis

Data collection continued until thematic saturation was reached, meaning that no new substantive themes emerged in the final interviews, suggesting that additional data collection was unlikely to generate novel conceptual insights. The interviews were conducted in both Arabic and English depending on participants’ preferences, allowing them to express themselves more comfortably. All audio recordings were transcribed manually by the researcher to maintain the authenticity and accuracy of participants’ accounts.

The interview transcripts were analyzed using thematic analysis within an interpretive and iterative framework (Braun and Clarke, 2006). Trustworthiness was enhanced through multiple strategies, including iterative coding, prolonged engagement with the data, and peer discussions among the research team to enhance reflexivity and analytical consistency. To enhance analytic rigor, decisions regarding aspects of coding such as density, frequency, size of data pieces to be coded (Elliott, 2018) were made collaboratively.

Initial coding was reviewed and then discussed among researchers to resolve discrepancies, refine code definitions, and reach consensus on coding decisions and category development.

Thematic analysis identified frequent patterns, while narrative analysis captured the tone, expressions of participants’ experiences (see Table 2 for an overview of the social media use and emerged themes). First, segments of the interviews that appeared to be relevant to the research questions were identified and coded (e.g., online community, family bond, religious purposes, stress, emotional support). These codes were grouped into broader categories and distilled into themes such us sense of belonging, cultural learning. Next, the transcripts were re-examined to make sure the codes and themes were interpreted in the appropriate contexts. The analysis then examined recurring intersections between social media gratifications and acculturation strategies. These intersections were organized into a thematic matrix (Table 3), which illustrates the patterns observed across participants’ accounts. Finally, selected anonymized quotations were used to illustrate the themes; ensuring participants’ confidentiality.

**Table 2:** Use of social media platforms and emerged themes.

RQs	Themes	Contextual definition	Exemplary quotes from the interviews
RQ1	Sustaining transnational family connection	Using platforms allows to stay connected and sustain emotional bonds with family.	*“Social media helps me connect with my family and check on them, and make sure they are doing well. Through Imo video calls, I can talk to and see my mom, dad, sister, and friends daily”*.
	Social media platforms as tools for cultural adjustment	Communicating with fellow workers online for encouragement or advice.	*“The groups that I follow support us as Ethiopian domestic workers in the UAE. When someone needs money, or someone is sick, or does not have work yet, these groups cooperate to help them.”*
	Digital collectivism for communal care	Search for sisterhood to help one another	*“I have groups in the UAE. If, for example, my mother gets sick, I send a message to the group to make duʿāʾ.”*
RQ2	Religious connection and gratification	Feeling part of an online religious community.	*“I like to watch YouTube to listen to the Qur’an, and listen to the rules of the Qur’an while I am cooking.”* *“I want to be a strong Christian preacher, so I watch videos on TikTok and YouTube.”*
	Learning for cultural norms and workplace expectations	Using social media to learn language, religion, norms, and news.	*“Social media helps me with my work. I see people teaching us how to work in the UAE, and I feel it is useful because I can ask for help or advice. I use TikTok to watch several things and follow anything helpful; I also use WhatsApp to communicate with people here.”*
	Information-seeking to prevent mistakes	Seeking information that helps with workplace tasks.	*“Social media helps me avoid misunderstandings in my workplace. When I want to know something, I search for it on social media.”*
	Mistrust and selective engagement with platforms	Exercising caution and selecting particular platforms	*“I do not like Instagram too much, I do not talk, but I see people’s posts. I see on TikTok, but I do not enter. I only listen and see. Maybe they lie, maybe they do not lie. I only see, I do not talk.”*

**Table 3:** Relationship between social media gratifications and acculturation strategies in migrant digital practices.

Gratification type	Evidence in the data	Acculturation strategy
Emotional and relational	Daily video calls, homework help, emotional reassurance from family (P1, P9)	Separation (Ethnic maintenance): sustained orientation toward home culture and strong transnational family bonding.
Social	WhatsApp prayer groups, mutual aid networks, collective duʿāʾ (P2, P4, P11)	Separation within buffering effects: strong diaspora reliance, with limited host-society contact
Informational and cognitive gratifications	Learning ironing techniques, Emirati customs, workplace expectations (P3, P7)	Integration: acquiring of host-culture knowledge and workplace competencies while maintain home cultural ties.
Spiritual	Watching Emirati prayer videos, strengthening own faith practices (P4, P6, P8)	Selective integration: adopting host-culture discipline while reinforcing home religious identity
Technological and affordance-related gratifications	Passive scrolling and low-engagement use of platforms, avoidance of interaction (P12)	Marginalization: minimal active participation, limited cultural anchoring in both home and host contexts due to disengaged use patterns.

## 4 Results

Overall, the analysis clearly shows that social media platforms help this sample of African domestic workers navigate their environments and workplace tasks; however, there is also evidence that social media platforms may further isolate them from the outside environment. Thus, while social media platforms may push them into an isolated “online cocoon” they also serve as the primary bridge to the outside world.

RQ1 focuses on how collectivistic values of this sample of African domestic workers shape their social media practices. Compared to individualism, collectivism emphasizes interdependence, group harmony, and family ties (Hofstede, 1980, 2011; Singelis et al., 1995). These collectivistic values may significantly influence how participants engage with social media platforms during their acculturation process. Thus, individuals from collectivistic cultures are more likely to use social media to maintain close relationships with peer domestic workers in the host country and family back home, in transnational contexts (Madianou and Miller, 2012). Further, by facilitating participation in diasporic support networks, social media may function as a space for sustaining emotional bonds and sharing collective experiences. It is likely that participants prioritize relational communication, group-based identities, and culturally shared content online.

### 4.1 Sustaining transnational family connection

Social media is deemed a lifeline linking migrant workers to their home countries. This connection allows them to share their experiences and knowledge about their work and living environments (Golan and Babis, 2019). Social media platforms also remind migrant workers of their cultural roots as they adapt to the host country’s culture. This digital engagement enhances migrant workers’ mental well-being, as supported by recent research on social media’s impact on migrant experiences (Sharmila and Mohamed Sahul Hameed, 2023). Further, communication via social media platforms can reinforce a sense of belonging to their children and families far away. Most participants mentioned the role of social media platforms in connecting with their families. For example, P1 mentioned:

*“Social media helps me connect with my family and check on them, and make sure they are doing well. Through Imo video calls, I can talk and see my mom, dad, sister, and friends daily.”*

Rather than indicating a full integration into the host society, P1expressed a form of transnational belonging, where she remains anchored in her home context. In the context of domestic work, this mediated connection, operates as both a communication tool and a form of transnational fields of connections where they receive updates from family members back home. The visual and emotional proximity that social media platforms provide can be a source of reassurance for domestic workers. The emotional and practical gratifications from using social media were apparent among the participants. For example, P9 expressed:

*“Sometimes in the evenings, my kids call me if they have homework: Mama, we have this, then I go on Google if I do not know it and tell them. I teach them online, and I use social media to talk to them.”*

The quote from P9 illustrates how social media use in transnational parenting is driven by multiple, overlapping gratifications rather than simple communication needs. Her statement reflects a strong informational and cognitive gratification, where digital platforms are actively used to acquire knowledge needed to fulfil parental responsibilities. In this sense, social media and search engines function as problem-solving tools that enable her to meet her children’s immediate educational needs despite physical separation. At the same time, the interaction also reflects emotional and relational gratifications, as digital communication allows the maintenance of maternal presence and responsiveness across distance. The mother’s engagement is not limited to information transfer but extends to sustaining emotional reassurance and continuity in the parent–child relationship. This aligns with UGT’s assumption that users actively select media to satisfy both instrumental and affective needs. Her sense of responsibility drives her to use technology to stay involved, showing that social media goes beyond just being a tool for reaching out to her family members, enabling her to experience and express her multiple identities, including her role as a mother, while living as a migrant worker. This highlights how transnational family connection is maintained not only through communication frequency, but through the ongoing reconstitution of maternal identity via digital mediation, where platforms enable both emotional attachment and functional caregiving across borders. Thus, social media becomes a multifunctional gratification space where cognitive (helping with homework), emotional (maintaining maternal bonding), and social (sustaining family connectedness) needs converge.

Many participants expressed similar experiences, using social media not only to maintain connections with their families, but also through layered gratification structure in which digital platforms are strategically mobilized to informational, emotional, and relational needs simultaneously.

### 4.2 Leveraging social media platforms for cultural adjustment

In collectivist societies, people tend to define their identity through their relationships with family, community, or social networks (Hofstede, 1980). In this context, social media platforms are the tools to form new relationships in the host country, assisting African domestic workers in navigating its cultural environment. For example, engaging in online support groups tends to reduce social isolation among Ethiopian domestic workers and provide guidance about living and working in a different culture (Choy et al., 2022). In this sense, social media becomes an informal resource that supports African domestic workers’ early stages of settlement and cultural adjustment. Such online interactions with others in similar working conditions and cultural background can facilitate domestic workers’ adaptation to the workplace and the broader cultural environment of the host country. In particular, Ethiopian domestic workers often perceive these platforms that connects them to both their cultural identity and practical knowledge about life abroad. African domestic workers exchange information about employment conditions, legal issues, and everyday coping strategies, which can reduce uncertainty in unfamiliar cultural settings. For example, participants described how online networks provide collective support among Ethiopian workers:

*“The most popular app I use in my free time is TikTok. I like to see videos about my country, Arabic videos, and Emirati cuisines. I see Ethiopian people cooking the Emirati food, and teaching us about many things”. (P2)*

This participant learns about local customs, cuisines, and practices, which helps her understand and comply with an institutionalized work environment. Beyond this individual adjustment, social media provide collective support network to adapt to workplace expectations.

*“The groups that I follow support us as Ethiopian domestic workers in the UAE. When someone needs money, or someone is sick, or doesn’t have work yet, these groups cooperate to help them.” (P4)*

These narratives suggest that these digital communities’ function as a form of collective solidarity, or a virtual sisterhood network that offers emotional, practical, and financial support, such as job-seeking, advising others, and practical help through social media. This emphasis on cooperation reflects the collectivist values, where shared group members feel a shared responsibility toward one another. In this sense, social media becomes a community space that reproduces offline cultural norms in digital environment.

### 4.3 Recreating community: social media and collective support

Social media also serves as a space where African domestic workers maintain the collectivist cultural values that shape their social relationships. In collectivist cultures, the identity of the “self” is strongly embedded in group belonging, mutual responsibility, and communal care (Hofstede, 1980). While forming and maintaining physical relationships are often difficulty, digital platforms provide online spaces where African domestic workers recreate familiar patterns of solidarity and shared moral support among domestic workers with the same or similar cultural background. Through social media platforms such as WhatsApp, Telegram, and TikTok communities, African domestic workers maintain strong interpersonal and group ties that resemble extended family structures (Komito, 2011). These networks are not only geared toward learning about the host society, but also toward sustaining their collective identity and emotional belonging within the migrant community. African domestic workers frequently engage in practices such as collective prayer, moral encouragement, and expressions of empathy when members face personal or family difficulties. Participants describe how these digital communities provide spiritual support:

*“I have WhatsApp groups from Saudi Arabia, Kuwait, and the UAE, like Abu Dhabi. I join these groups to make me close to them and make me feel comfortable and stay more motivated to work here in the Emirates and gives me energy to continue working and to be strong". (P2)*

Participant join groups that function as transnational social spaces that reduce isolation and create a sense of community. Other participant extended the role of these digital networks beyond emotional support to include spiritual and collective practices.

*“I have groups in the UAE. If, for example, my mother gets sick, I send a message to the group to make duʿāʾ (supplication), and they pray for her.” (P11)*

These quotes describe how the online community groups reflect home values of kinship and share responsibility, especially in vulnerability cases. This also reflects that the collectivistic communal care from her peers on digital groups in the UAE, extending beyond practical communication to include emotional and spiritual support and group prayer. This confirms the mutual help and support she expects from her digital peer networks and illustrates the collectivist cultural values that shape these workers. The practices of digital collective prayer (duʿāʾ) enhance the sense of faith and belonging, and duty; they also reinforce the moral support that is rooted in their culture. From an acculturation standpoint, these behaviours illustrate the strategy of cultural integration (Berry, 1997), where migrant workers adapt digital tools from the host country while combining them with their culture and practices. African domestic workers are seeking emotional and relational support and social belonging, which reflects how digital platforms help them acculturate to the UAE culture. However, while social media networks strengthen solidarity within the migrant community, they may also reinforce inward-looking social ties that limit interaction with the broader host society.

Overall, the findings show that African domestic workers’ use of social media platforms reflects a dynamic interplay between inherited collective cultural values and the needs of their lived experiences in the host country, which demonstrates how collective cultural orientations are permeated in their social media use. Social media platforms enable African domestic workers to sustain transnational family ties, participate in diasporic support networks, and exchange information about living and working abroad. Social media also facilitates communal care practices such as collective prayer, emotional encouragement, and mutual assistance during times of difficulty. Through such interactions, social media spaces serve as extensions of collectivistic values by promoting group belongings and solidarities, and at the same time providing opportunities for cultural adaptation of the individual in the host society.

RQ2 explores how African domestic workers use social media to satisfy social, emotional, and informational needs that support their acculturation in the UAE. The findings show that participants actively select social media platforms to obtain different forms of gratification (Katz et al., 1973; Sundar and Limperos, 2013). Overall, social media satisfies multiple needs that facilitate African domestic workers’ adaptation and integration into the host society.

### 4.4 Digital religious practices and spiritual support

Social media platforms serve as a retreat, allowing migrant workers to participate in prayer, worship, and religious sermons. In this sense, social media helps reduce cultural and religious barriers, allowing them to express their faith and religion freely (Brinkerhoff, 2009). Exposure to religious practices from both the origin and host countries encourages spiritual and social sustainability, as Christian and Muslim African domestic workers share prayers and spiritual content that reinforce their emotional and spiritual identities within digital religious communities. Despite their diverse cultural backgrounds, participants find common values, culture, and beliefs that bind them together, forming a blended mixture that transcends race and language through platforms such as WhatsApp, Telegram, and Imo. This aligns with Berry’s (1997) theoretical perspective on acculturation, which illustrates that social integration can occur through spiritual practices. Likewise, UGT (Katz et al., 1973; Sundar and Limperos, 2013) provides insights into workers’ social media use not only for entertainment or knowledge, but also for religious, social, and spiritual gratifications, as faith-based content offers a source of comfort, safety, and an enhanced sense of belonging, stability, and social cohesion for these African domestic workers. For examples,

“*I used to read the Qur’an a lot, but for the last one and a half years, I haven’t read in a long time. I am upset because, although I had free time, I didn’t know why I didn’t read. Now I read” (P5)*

This statement illustrates how digital platform facilitate spiritual continuity, even after periods of disruptions. The return to reading the Quran indicates a reconnection with the spiritual identity and suggest that use of social media may help preestablishing faith-based routines.

*“I like to watch YouTube to listen to the Qur’an, and listen to the rules of the Qur’an while I am cooking.” (P6)*

Listening to Quran while performing daily tasks shows that religious practices and spiritual engagement can be adapted to the constraints of workplace, where time and space for formal worship may be limited. This reflects a form of religiosity, where digital platform allows maintaining routines despite temporal or physical challenges. Campbell and Tsuria (2021) explain that digital spaces allow believers to negotiate their religious belonging across borders, supporting the preservation of faith in transnational contexts. In this sense, social media not only facilitate access to religious content but also contribute to the emotional well-being of among migrant workers by bridging home and host environments.

In particular, platforms such as YouTube, TikTok, and WhatsApp make it easier to understand and share religious knowledge among peer workers. For example, P4 notes:

*“I learned through social media the traditions of emirates and the way they pray and go to the mosque, which motivated me as a Christian to strengthen my religion, because, as a Christian, I don’t pray every day, but by watching Emiratis’ videos, it helped me to pray more.”*

This reflects selective integration (Berry, 1997), where P4 who was exposed to the host culture’s visible religiosity selected these practices as a resource that reinforces, rather than replaces, her original spiritual identity, satisfying spiritual gratifications.

By observing the Emirate’s religious practices on social media, the participant felt inspired to strengthen her own faith. P4’s comment demonstrates that social media may function as a tool of cultural learning and spiritual growth, allowing migrant workers to integrate host country practices with existing religious routines. P8 also states that she follows Christian sermons and recitations via YouTube and TikTok, attributing this to her strong commitment to her faith and considering it a source of spiritual nourishment. In the same context, P12 expressed her love for the Christian traditions she follows through TikTok to promote her religious identity, whether by listening, posting photos, or interacting with digital communities that share the same values.

### 4.5 Learning cultural norms and workplace expectations

Social media shapes the work performance of African domestic workers and facilitates their integration while they are in the UAE. When they share videos, tips, and join groups, this content enhances their work practices and helps them adjust to their workplace culture and norms. Digital platforms function as informal learning spaces that provide essential resources, knowledge, and experience for users (Komito, 2011; Leurs, 2015). Additionally, these platforms help them understand cultural norms and navigate challenges in their workplace. For example, YouTube, TikTok, and WhatsApp are tools that help African domestic workers learn new skills and adapt to the workplace expectations (Akter et al., 2024):

*“Social media helps me with my work. I see people teaching us how to work in the UAE, and I feel it is useful because I can ask for help or advice. I use TikTok to watch several things and follow anything helpful; I also use WhatsApp to communicate with people here”. (P3)*

P3 discloses her usage of social media, particularly TikTok, to learn new elements that contribute to the development of her working skills. The way she talks suggests she has limited learning instructions, which reveals a fear of asking her employees for help and guidance. Using social media as a learning platform reflects a repeated pattern of gratification among domestic workers on TikTok. P3 demonstrates the valuable role of having online peers to provide the help or advice she may need, showing how she selects platforms that match these needs. Her ability to seek advice and support from her peers on social media makes it easier for her to adapt in the workplace. This illustrates how digital platforms provide domestic workers with access to the knowledge that bridges the gaps in their workplace expectations, such as providing skills and practical information. Moreover, they can decrease social pressure and barriers in communication with their employers in the workplace. These findings suggest that social media is a critical informal teaching system, which helps participants adapt to the UAE workplace culture and enhance their competence (Komito, 2011; Leurs, 2015).

Participants use social media to seek specific information that helps them adapt to their workplace in the UAE. They also use these platforms as a primary resource for information such as salaries, rights, and labour laws. From an information-seeking perspective, social media offers clear guidance that helps migrant workers reduce workplace confusion (Katz et al., 1973). This enables them to reduce mistakes and handle workplace issues and expectations. For example, P7 mentions:

*“Social media helps me to avoid misunderstandings in my workplace. When I want to know something, I search for it on social media. For example, I iron clothes in wrong way because I didn’t know that some dress requires dry cleaning, other dress requires hand washing, so I searched the internet using social media to know what the mistake is.” (P7)*

P7 chooses to learn from social media to avoid mistakes in her workplace. In her example, she realizes the error after it occurred, which makes her turn to social media to understand the instructions properly. She utilizes social media as a learning tool to optimize her work and gain more knowledge (Komito, 2011; Leurs, 2015). Participants proactively use social media to learn cultural expectations, household chores, and nuances of garment care; these actions help them reduce conflict in the workplace, enhance their efficiency, and demonstrate cultural adaptation to Emirati families. Overall, social media assists workers in minimizing risk, maintaining stability, and enhancing work satisfaction, which suggests that social media provides gratification for their needs.

### 4.6 Selective engagement and digital mistrust

While social media platforms can be used to reduce linguistic and cultural barriers, participants’ engagement is selective rather than unconditional. Some carefully evaluate information without participating, reflecting awareness of potentially misleading or fabricated content. To mitigate these risks, participants often adopt passive consumption strategies to avoiding commenting, posting or interacting with other users while still using these platforms, to observe and learn. For example, P12 mentions:

*“I don’t like Instagram too much, I don’t talk, but I see people’s posts. I see on TikTok, but I don’t enter. I only listen and see. Maybe they lie, maybe they don’t lie. I only see, I don’t talk”. (P12)*

P12’s quote illustrates selective engagement does not indicate isolation from the digital world, but rreflects a strategic mode of participation. The participant’s preference for “seeing” without “talking” suggests a form of digital self-protection, whereby limited interaction serves as a coping strategy to avoid exposure to misinformation, judgment, or emotionally distressing content. In this sense, passive engagement becomes a way to maintain access to social media benefits while minimizing potential risks. This pattern can be understood as a marginalization strategy, in which constrained users adopt low-visibility practices to navigate uneven digital environments and reduce potential harm. It also aligns with literature on “lurking” behaviors (Hong et al., 2023), where users engage in observational forms of participation that sustain social awareness and identity work without active contribution or interaction.

Previous research suggests that some migrant workers intentionally exercise limited digital engagement to maintain psychological balance in an environment that may be stressful and unsafe for them (Alencar, 2018). In this sense, social media use reflects not only opportunities for connection and acculturation, but also exposure to digital stressors, uncertainty, and mistrust, highlighting its dual and sometimes contradictory role in shaping the acculturation process. Rather than engaging fully or withdrawing entirely, participants in this study demonstrate selective and cautious patterns of engagement, actively filtering content and avoiding interactions with potentially negative or distressing material. Building on this perspective, the selective engagement observed among participants in this study can be interpreted as a form of informal self-regulation, where individuals consciously limit exposure to potentially distressing or negative content as a coping strategy. This aligns with the broader literature (eg., Reinecke et al., 2022) suggesting that media affordances and social dynamics shape users’ capacity to regulate their engagement, ultimately influencing whether social media functions as a resource for adaptation or a source of psychological strain.

### 4.7 Gratification-acculturation patterns in migrant digital practices

The findings reveal four interrelated gratification-acculturation patterns shaping migrant workers’ digital practices. First, pure separation emerges when relational and emotional gratifications are dominant, with limited engagement in host-society content and a sustained orientation toward home-based ties. Second, instrumental integration is evident when informational and learning gratifications are actively pursued—particularly related to work and cultural adaptation—while emotional anchoring remains primarily within the home culture. Third, selective or hybrid integration reflects a more layered form of engagement in which spiritual gratifications are drawn from both home and host contexts, enabling participants to incorporate aspects of host cultural discipline while reinforcing their own religious identity. Finally, protective disengagement characterizes patterns of passive scrolling and digital mistrust, where minimal interaction functions as a coping strategy to maintain psychological safety, though it may inadvertently constrain deeper social integration.

The findings for RQ2 illustrate that social media platforms are an essential resource for learning, adapting, and generating mutual family and cultural connections during the migration period. Participants do not passively consume content. They actively engage with platforms that fulfil their cognitive, emotional and social needs, enhancing their sense of inclusion. Digital observational learning provides opportunities that foster a better understanding and appreciation of the host country’s religious culture.

The findings for RQ1 and RQ2 reveal the significant role of social media in shaping participants’ acculturation and everyday lived experiences in the UAE.

Collectivist cultural orientations strongly structure platform use, particularly in maintaining transnational family ties, diasporic support networks, and practices of mutual care, including financial, emotional and spiritual support. Consistent with UDT (Katz et al., 1973; Sundar and Limperos, 2013), findings also show that participants actively use social media to gain religious, informational, and learning gratifications. Thus, social media functions as both an online support system and an informal learning space that facilitates participants’ adaptation to the host society. However, while social media enables workers to maintain relationships with their roots and culture and meet their needs, the findings also suggest dysfunctions in social media use. For example, some participants work in isolation, and social media may become their only connection to the outside world. That is, social media helps them acculturate into society, but their dependency on it may discourage seeking active external, face-to-face connections.

## 5 Conclusion

This study demonstrated that for this sample of African domestic workers in the UAE, social media is more than a communication tool; it plays a significant role in shaping their acculturation experience. As participants balance maintaining ties with home culture while engaging with the host culture, platforms such as Imo, TikTok, and WhatsApp serve as important tools for maintaining emotional connections, offering meaningful psychological support through daily video calls, homework support, and constant updates on family life, thereby mitigating feelings of alienation and homesickness. Participants explained that they utilize digital platforms for multiple reasons, including religious practices, such as listening to religious recitations or sermons. This reflects a selective integration strategy, where workers choose specific ways to engage with the host culture while using social media to strengthen their original Christian faith, thereby balancing cultural adaptation with preservation of their spiritual identity. At the same time, it is important to recognize the diversity of African countries and cultures, which means experiences may vary across nationalities, religions, and contexts.

The use of social media strengthens domestic workers’ sense of collectivistic belonging. Platforms such as TikTok, Facebook, and YouTube serve as informal learning spaces, allowing domestic workers to acquire practical knowledge about workplace norms and cultures, thereby enhancing confidence in fulfilling their duties. Some participants actively participate in online sisterhood networks and faith-based communities that offer emotional reassurance, financial assistance, and spiritual guidance. These digital spaces compensate for restricted mobility and limited offline interactions in the host country.

The study also reveals the dual nature of social media platforms for domestic workers. While social media platforms create digital “shelters” of support and collectivist mutual aid, they can also intensify digital stress. Defined as the cognitive demand emerging from the ubiquity and use of digital technologies, digital stress is a growing concern in the literature (Hefner and Vorderer, 2016). To cope with this stress, some participants engage in cautious and largely observational strategies, limiting their active participation, to protect themselves from misinformation, online criticism, and potential emotional harm. In this way, social media serve as tools not only for cultural learning and social connection but also for managing the boundaries of cultural adaptation. Through these selective strategies, meanings are continuously reshaped through everyday digital exchanges (Chen et al., 2025). These dynamics suggest that acculturation is negotiated not only through physical encounters in workplaces and public spaces, but also through algorithmically mediated interactions, group chats, and content consumption practices. These findings align with the UGT, illustrating that domestic workers are not passive media consumers but active agents who selectively engage with platforms that provide emotional reassurance, social support, and practical guidance relevant to their everyday challenges. Theoretically, the findings extend UGT by demonstrating how social, emotional, informational, and spiritual gratifications intersect in the digital lives of female domestic workers. The study also nuances Berry’s (1997) acculturation framework by revealing hybrid patterns of integration, separation, and partial integration enacted through selective engagement with social media.

For some domestic workers, continuous engagement with home-country communities and content strengthens home-country identity but may slow deeper interaction with Emirati social environments. Thus, the study illustrates social media as an essential but ambivalent resource that supports cultural adaptation, identity negotiation, and well-being, while also carrying risks of isolation, and exposure to potentially misleading content. The findings suggest that policymakers, should support digital programs that support cultural exchanges between domestic workers and host communities and develop guidelines, training, or resources for digital literacy and emotional support tailored to migrant workers. Because some participants expressed mistrust and engaged in passive consumption of content, targeted digital literacy initiatives could help migrant workers critically evaluate online information, recognize misinformation, protect privacy, and safely engage with digital communities.

This study has several limitations that should be acknowledged. First, the study’s small sample size limits the generalizability of the findings. The research relied on in-depth interviews with twelve African female domestic workers. Specifically, a snowball sampling method was used to recruit participants, mainly from Ethiopia, due to the difficulty of accessing a population scattered across the study region. Thus, the sample does not fully represent the diversity of African domestic workers in the UAE. Additionally, participants represent only a small number of nationalities, and Africa is a vast and diverse continent, so the results cannot be considered representative of all African domestic workers in the UAE. Second, interviews were conducted in Arabic and English to accommodate participants’ preferences. This may have affected participants’ ability to fully express complex emotions, cultural experiences, or nuanced reflections on their acculturation processes. Although privacy protocols were fully implemented and explained, the sampling strategy may have introduced potential biases that should be acknowledged. Participants were recruited through employer-mediated access, which may have influenced both participant selection and the nature of their responses. Employer involvement in facilitating access could have shaped perceptions of surveillance, dependency, or lack of full anonymity, potentially affecting participants’ willingness to disclose critical or negative experiences. In such contexts, participants may have engaged in self-censorship or selective disclosure, particularly regarding sensitive workplace or social media practices. In addition, snowball sampling may have reinforced homogeneity in the sample by relying on existing social or professional networks. These dynamics may have contributed to a tendency to a socially desirable responses, consistent with “everything is perfect” (Bergen and Labonté, 2020) effect, a well-known limitation in qualitative research.

Third, the research was conducted in one emirate within the UAE; therefore, the findings may not be transferable to other parts of the country or global contexts where social and labor conditions differ.

Future research could benefit from including domestic workers from a broader range of African countries. Comparative studies across different emirates or host countries could allow for a deeper understanding of how cultural background and labor conditions shape social media use and acculturation strategies. A more critical engagement with digital inequalities and platform governance would further enrich the analysis and represents an important avenue for future research. Second, future studies may benefit from adopting longitudinal research designs to capture how digital practices and acculturation processes evolve over time. Examining individual workers’ experiences across different stages of migration could offer insights into how sustained social media use influences identity negotiation, emotional well-being, and workplace adaptation. Third, incorporating the perspectives of employers, policymakers, or labor institutions could provide a more holistic understanding of how digital media intersect with labor governance and migrant well-being.

In conclusion, this study illustrates how social media functions as a critical acculturation resource for this sample of African domestic workers while simultaneously operating as cultural, emotional, and spiritual spaces through which workers maintain transnational ties and navigate life in the host society. At the same time, the findings reveal the paradoxical nature of social media, demonstrating how it can both facilitate adaptation and contribute to isolation, digital stress, and limited interaction with the broader host environment. Theoretically, the study extends UGT by showing how social, emotional, informational, and spiritual gratifications intersect in the digital lives of migrant domestic workers. By examining the lived experiences of an underrepresented population, this study fills an important gap in migration and communication scholarship.

## Data Availability

The raw data supporting the conclusions of this article will be made available by the authors, without undue reservation.
